# Transcriptional supercoiling boosts topoisomerase II-mediated knotting of intracellular DNA

**DOI:** 10.1093/nar/gkz491

**Published:** 2019-06-05

**Authors:** Antonio Valdés, Lucia Coronel, Belén Martínez-García, Joana Segura, Sílvia Dyson, Ofelia Díaz-Ingelmo, Cristian Micheletti, Joaquim Roca

**Affiliations:** 1 Molecular Biology Institute of Barcelona (IBMB), Spanish National Research Council (CSIC), Barcelona 08028, Spain; 2 Scuola Internazionale Superiore di Studi Avanzati (SISSA), 34136 Trieste, Italy

## Abstract

Recent studies have revealed that the DNA cross-inversion mechanism of topoisomerase II (topo II) not only removes DNA supercoils and DNA replication intertwines, but also produces small amounts of DNA knots within the clusters of nucleosomes that conform to eukaryotic chromatin. Here, we examine how transcriptional supercoiling of intracellular DNA affects the occurrence of these knots. We show that although (−) supercoiling does not change the basal DNA knotting probability, (+) supercoiling of DNA generated in front of the transcribing complexes increases DNA knot formation over 25-fold. The increase of topo II-mediated DNA knotting occurs both upon accumulation of (+) supercoiling in topoisomerase-deficient cells and during normal transcriptional supercoiling of DNA in *TOP1 TOP2* cells. We also show that the high knotting probability (*P^kn^* ≥ 0.5) of (+) supercoiled DNA reflects a 5-fold volume compaction of the nucleosomal fibers *in vivo*. Our findings indicate that topo II-mediated DNA knotting could be inherent to transcriptional supercoiling of DNA and other chromatin condensation processes and establish, therefore, a new crucial role of topoisomerase II in resetting the knotting–unknotting homeostasis of DNA during chromatin dynamics.

## INTRODUCTION

**Figure 1.**
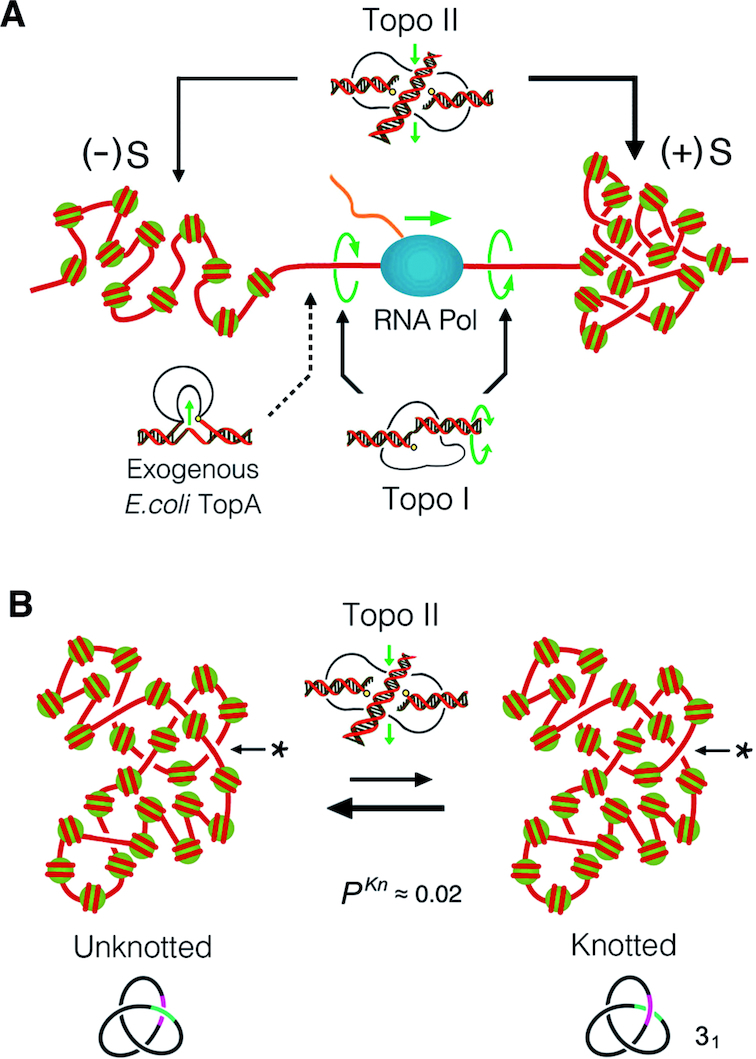
Topoisomerase activities that modulate supercoiling and knotting of intracellular DNA. (**A**) Both topo I and topo II can relax the (+)S and (−)S of DNA generated, respectively, in front of and behind the transcribing complex. The DNA-swiveling mechanism of topo I performs nearby the RNA polymerase, whereas the DNA cross-inversion mechanism of topo II performs at DNA crossings formed within nucleosomal fibers. Exogenous expression of *Escherichia coli TopA* relaxes (−)S only and thereby leads to the accumulation of (+)S, when topo I and topo II are inactivated. (**B**) The mechanism of topo II can produce and remove DNA knots by inverting juxtapositions of DNA linker segments (*) within nucleosomal fibers. The scheme illustrates the formation and resolution of a trefoil knot (31) in a circular minichromosome of ∼25 nucleosomes, whose DNA knotting probability (*P^Kn^*) *in vivo* is ≈ 0.02.

During DNA transcription, rotation of the duplex relative to the RNA polymerase produces positive supercoiling of DNA ((+)S) in front of the transcribing complex and negative supercoiling ((−)S) behind it (1,2). In eukaryotic cells, topoisomerases I and II (topo I and topo II) facilitate RNA synthesis by relaxing the transcriptional supercoiling of DNA (3,4). Topo I produces transient DNA nicks to allow swiveling of the duplex and thus relaxation of (+)S and (−)S (5). Topo II produces transient DNA double-strand breaks and passes across them another segment of DNA (6). This DNA cross-inversion mechanism allows the relaxation of (+)S and (−)S, as well as the elimination of the DNA intertwines that arise during chromosome replication (3,4) (Figure 1A).

Although either topo I or topo II suffices to relax (+)S and (−)S *in vivo*, fine-tuning of chromosomal DNA topology requires the interplay of both topoisomerases with chromatin architecture (7,8). Whereas topo I relaxes efficiently naked DNA regions, topo II is more proficient in chromatinized DNA (9). Accordingly, topo I is recruited in adjoining DNA-transcribing complexes, where nucleosomes are transiently disrupted and spinning of the duplex is fast (10), whereas topo II is recruited mainly in intergenic regions, away from open reading frames (11,12) (Figure 1A).

The chromatin architecture also determines the dissipation and relaxation rates of (+)S and (−)S. The organization of cellular chromosomes into topological domains and the rotational drag of chromatin fibers delay the diffusion and cancellation of DNA supercoiling waves (13,14). Accordingly, domains with different levels of (+)S and (−)S have been mapped within transcriptionally active regions throughout chromosomes of yeast (15), *Drosophila* (16) and human cells (17,18). Detection of these (+)S and (−)S domains also indicates that transcriptional supercoiling of DNA is not instantaneously relaxed or dissipated. In this respect, the activity of intracellular topoisomerases has been found to relax (+)S domains faster than (−)S domains (19). Since (+)S and (−)S are generated at similar rates during DNA transcription, their asymmetric rate of relaxation produces a homeostatic excess of (−)S, thereby overcoming the necessity of a DNA-unwinding topoisomerase (i.e. bacterial DNA gyrase) in eukaryotic cells (19).

Efficient relaxation of (+)S facilitates transcriptional elongation, and the activity of either topo I or topo II can fulfil this task. However, studies in mouse and human cells demonstrate that a combined function of topo I and topo II is required for proper transcription elongation, particularly during the synthesis of long RNA transcripts (20–22). This requirement affects long genes involved in neural development (20) and synaptic function (23) and linked to autism (24). Likewise, in yeast cells, proper transcription of long genes requires both topoisomerases and becomes blocked when topo II is inactivated (25). Strikingly, this stalling of RNA polymerases can be rescued by exogenous expression of type-2 (topo II), but not type-1 (topo I) topoisomerases (25). These observations led us to hypothesize that the constraints impairing transcription elongation could be DNA knots (i.e. intramolecular entanglements of DNA), since only type-2 topoisomerases can knot–unknot duplex DNA (3,6). Supporting this hypothesis, *in vitro* studies have shown that DNA knots are able to impair DNA transcription (26). In this respect, we recently uncovered that DNA knots are present in intracellular chromatin (27). Topo II-mediated knotting of DNA occurs within stretches of ∼25 nucleosomes with a probability of ∼0.02 (Figure 1B). These findings opened up the question of how the knotting probability of intracellular DNA affects or is affected by genome activities and chromatin architecture.

Here, we examine how the occurrence of intracellular DNA knots is affected by transcriptional supercoiling of DNA. We show that (+)S increases topo II-mediated knotting of DNA over 25-fold and that this increase is consequent to chromatin compaction. Our findings show that DNA knotting concurs normally with transcriptional supercoiling and other chromatin condensation processes, and establish therefore a new crucial role of topo II in resetting the DNA knotting–unknotting balance during the conformational transitions of intracellular chromatin.

## MATERIALS AND METHODS

### Yeast strains, plasmids and enzymes

All experiments were conducted in *Saccharomyces cerevisiae* strains JCW25 (*TOP1 TOP2*) and JCW28 (*Δtop1 top2–4*), which are derivatives of FY251 (*MATa his3-Δ200 leu2-Δ1 trp1-Δ63 ura3–52*). JCW28 carries the null mutation *Δtop1* and the thermosensitive mutation *top2–4* (28). When indicated, JCW25 and JCW28 were transformed with pJRW13, a plasmid that carries the *Escherichia coli TopA* gene under the constitutive p*GPD* yeast promoter (9). Circular minichromosomes YRp4 (27), YEp24 (27) and pYR121 (29) were amplified as bacterial plasmids in *E. coli* and used to transform *S. cerevisiae* following standard procedures. Topo I of vaccinia virus was purified from *E. coli* cells harboring the expression clone pET11vvtop1 as described previously (30). Topo II of *S. cerevisiae* was purified from yeast cells harboring the expression clone YEpTOP2GAL1 as described previously (31). The DNA-nicking endonuclease BstNB1 was purchased from NEB.

### Yeast culture and DNA extraction

Yeast cells were grown at 26°C in yeast synthetic media containing adequate dropout supplements and 2% glucose. Thermal inactivation of topo II was carried out during exponential growth (OD ≈ 0.8) by shifting cell cultures to 37°C for the indicated time periods. Activation of the *G*A*L1GAL10* promoter of pRY121 was performed by transferring the cells that grew in media containing 2% glucose into YP Broth media containing 2% galactose for 3 h. Before harvesting yeast cells, intracellular DNA topology was fixed as described previously (32) by quickly mixing the liquid cultures with one cold volume (−20°C) of ETol solution (ethanol 95%, 28 mM toluene, 20 mM Tris-HCl, pH 8.8, 5 mM EDTA). Fixed cells from a 25 ml culture were sedimented, washed twice with water, resuspended in 400 μl of TE (10 mM Tris-HCl, pH 8.8, 1 mM EDTA) and transferred to a 1.5-ml microfuge tube containing 400 μl of phenol and 400 μl of acid-washed glass beads (425–600 μm, Sigma). Mechanic lysis of >80% cells was achieved by shaking the tubes in a FastPrep® apparatus for 10 s at power 5. The aqueous phase of the cell lysates was collected, extracted with chloroform, precipitated with ethanol and dissolved in 100 μl of TE containing RNAse-A. Following 10 min of incubation at 37°C, DNA was precipitated with ammonium acetate and ethanol, and then dissolved in 40 µl of TE.

### DNA topology analysis by 2D-gel electrophoresis

To examine the Lk distribution of minichromosomes, 2D-electrophoreses of YRp4 and 2-micron circles were carried out in 0.8% agarose gels (20 cm × 20 cm) in TBE buffer (89 mM Tris-borate, 2 mM EDTA) plus 0.6 μg/ml of chloroquine at 50 V for 14 h in the first dimension, and TBE buffer plus 3 μg/ml of chloroquine, at 60 V for 8 h in the second dimension. 2D-electrophoreses of YEp24 and pRY121 were carried out in 0.6% agarose in TBE buffer plus 0.6 μg/ml of chloroquine at 30 V for 36 h in the first dimension, and TBE buffer plus 3 μg/ml of chloroquine, at 80 V for 4 h in the second dimension. To examine the DNA knots formed in the minichromosomes, their DNA were nicked with endonuclease BstNBI and loaded in a 20 cm × 20 cm agarose gel. 2D-electrophoreses of YRp4 were carried out in a 0.9% agarose gel in TBE buffer at 33 V for 40 h in the first dimension, and at 150 V for 3 h in the second dimension. 2D-electrophoreses of 2-micron circles, YEp24 and pYR121 were carried out in 0.6% agarose (2-micron) or 0.45% agarose (YEp24 and pYR121) in TBE buffer at 25 V for 40 h in the first dimension, and at 125 V for 4 h in the second dimension. 2D-gels were blot-transferred to a nylon membrane and probed with minichromosome-specific DNA sequences labeled with AlkPhos Direct (GE Healthcare®). Probe signals of increasing exposure periods were recorded on X-ray films. DNA knot probability (*P^Kn^*) was calculated as described previously (27), as the total fraction of nicked knotted DNA circles (irrespectively of the knot complexity) relative to the total amount of nicked DNA circles (knotted and unknotted).

### Numerical simulation of DNA knotting in modeled nucleosomal fibers

The YRp4 minichromosome was modeled as a ring made of 25 spherical beads of diameter *D*, each representing a nucleosome, and infinitely thin straight segments connecting the centers of neighboring beads, such that the free portion of a segment, of length *L*, represented a DNA linker. A Metropolis Monte Carlo scheme based on crankshaft moves was used to evolve the system, which was initially prepared in a circular arrangement. Excluded volume effects were introduced by assigning infinite energy to configurations with overlapping beads, and zero energy otherwise. The Monte Carlo moves allowed the linkers to cross so that the sampled space corresponded to torsionally relaxed and topology unrestricted minichromosomes. For different combinations of the *D/L* ratio in the [0:1] range, we collected 10^5^ uncorrelated conformations (i.e. picked at time intervals larger than the autocorrelation time of the radius of gyration radius, *R*_g_) that were topologically profiled by comparing the Dowker code of their 2D projections against tabulated values. The Knotscape software (http://pzacad.pitzer.edu/∼jhoste/hostewebpages/kntscp.html) was used for this purpose. As the basal *P^Kn^* ∼ 0.02 of YRp4 *in vivo* was recovered for *D/L* ∼ 0.47, the effect of compaction at this value of *D/L* was accounted for by keeping only configurations with relative gyration radius smaller than a threshold value, max*R*_g_, out of a more extensive set of ∼4 × 10^6^ uncorrelated conformers, which yielded an average (root mean square) gyration radius *R*_g_^0^/(*D* + *L*) of 1.63. Absolute writhe (|*W*r|) was computed by averaging the sum of the signed crossings (defined according to the right-hand rule after orienting the curve) over hundreds of projections. For each sampled conformation, *R*_g_*/R*_g_^0^ was averaged for different (binned) ranges of |*W*r|.

## RESULTS

### DNA knotting probability changes differently during (+) and (−) supercoiling of intracellular chromatin

As in previous studies, we used yeast circular minichromosomes to analyze DNA knot formation in intracellular chromatin (27). Since (+)S and (−)S cancel each other in circular DNA domains, we accumulated (+)S and (−)S separately to reproduce the conformations generated during transcriptional supercoiling of chromosomal DNA. We generated (+)S upon topo II inactivation in *Δtop1 top2–4* cells that constitutively expressed *E. coli TopA* (2). In these conditions, *TopA* relaxes the (−)S but not the (+)S generated during DNA transcription. Likewise, we generated (−)S upon thermal inactivation of topo II in *Δtop1 top2–4* cells. In these conditions, preferential relaxation of (+)S by residual topo II leads to the accumulation of (−)S (19) (Figure 1A).

**Figure 2.**
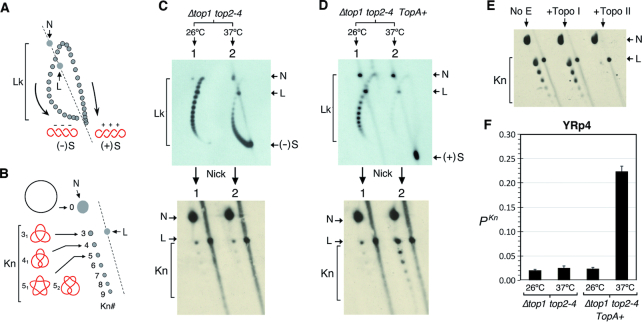
DNA knotting probability during (+) and (−) supercoiling of chromatin. (**A**) Lk distribution of DNA topoisomers (Lk) in a 2D-gel electrophoresis (first dimension, top to bottom; second dimension, left to right). Arrows denote Lk displacement upon increasing (+)S and (−)S of DNA; N, nicked DNA circles; L, linear DNA. (**B**) Relative position of unknotted (N) and knotted nicked DNA circles (*Kn*) in a 2D-gel electrophoresis. The velocity of knotted molecules in the first gel dimension (top to bottom) correlates with their number of irreducible of DNA crossings (*Kn*#). Knots 3_1_, 4_1_, 5_1_ and 5_2_ are depicted. (**C** and **D**) DNA topology of YRp4 in *Δtop1 top2–4* (C) and in *Δtop1 top2–4 TopA+* (D) cells. Cells were sampled at 26°C (lane 1) and following 120 min at 37°C (lane 2). (**E**) Incubation of the nicked DNA sample of (+)S YRp4 (no E) with topo I and topo II activities *in vitro*. (**F**) DNA knotting probability (*P^Kn^*) of YRp4 in the four conditions analyzed in panels (C) and (D). Data in panel (F) are presented as mean ± SD of three experiments.

Upon fixing the DNA topology of the minichromosomes *in vivo* (32), we examined the superhelicity of their DNA by means of 2D-gel electrophoresis (33). In these gels (Figure 2A), linking number topoisomers (Lk) of circular DNA distribute along an arch, in which Lk values increase in the clockwise direction. Accumulation of (+)S is thus denoted by a clockwise displacement of the Lk distribution, whereas increase of (−)S is denoted by a counterclockwise shift. To examine the presence of DNA knots in the supercoiled minichromosomes, we nicked their DNA to eliminate any supercoiling and conducted a different kind of 2D-gel electrophoresis (34). In these gels (Figure 2B), nicked DNA circles that contain knots move faster than the unknotted nicked circle, and their velocity correlates to the knot complexity (the number of irreducible DNA crossings of a knot, *Kn*#).

Figure 2C shows the DNA supercoiling (top gel) and DNA knotting (bottom gel) states of YRp4, a 4.5-kb minichromosome, in *Δtop1 top2–4* cells. Before topo II inactivation (lane 1), the Lk distribution reflected the negative supercoils that are normally constrained by native nucleosomes. Upon thermal inactivation of topo II (lane 2), accumulation of (−)S was evidenced by a counterclockwise shift of the Lk distribution. However, the signals of DNA knots did not significantly change with the generation of (−)S (bottom gel, compare lanes 1 and 2). Figure 2D shows the analogous experiment conducted in *Δtop1 top2–4 TopA+* cells. In this case, the typical Lk distribution constrained by native nucleosomes (top gel, lane 1) was shifted entirely clockwise after the thermal inactivation of topo II (lane 2), denoting the accumulation of (+)S in all the minichromosomes. Strikingly, in this condition, the signals of DNA knots increased markedly with the accumulation of (+)S (bottom gel, compare lanes 1 and 2). We corroborated that the increased signals were knots of double-stranded DNA by incubating the sample with topo I and topo II *in vitro*. As expected, only topo II was able to unknot the DNA and so reduce the increased signals (Figure 2E). Quantification of the DNA knot probability (*P^Kn^*) of YRp4 indicated that, prior to accumulation of (−)S or (+)S, *P^Kn^* was ∼0.02, similar to that previously observed in *TOP1 TOP2* cells (27). *P^Kn^* did not change significantly with (−)S, but increased ∼10-fold with (+)S (Figure 2F).

### (+) Supercoiling boosts DNA knotting probability and knot complexity

**Figure 3.**
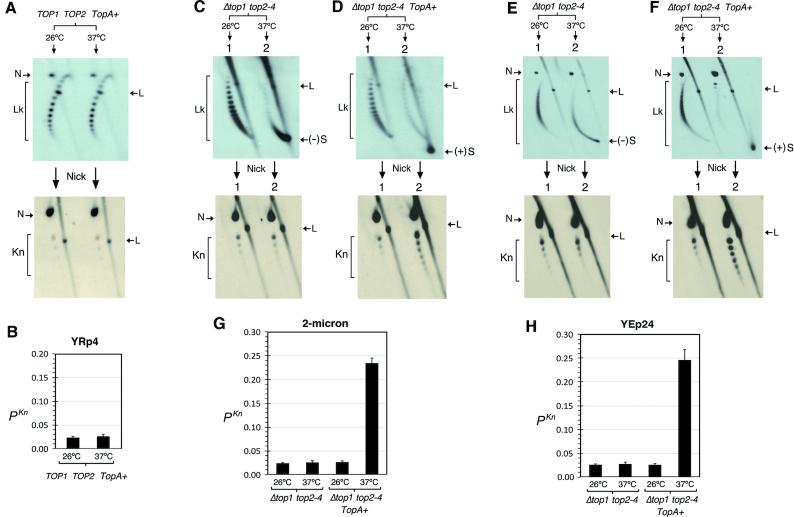
Increased knot formation is caused by (+) supercoiling of DNA. (**A**) DNA supercoiling and knotting of YRp4 in *TOP1 TOP2 TopA+* cells sampled at 26°C and following 120 min at 37°C. (**B**) *P^Kn^* of YRp4 in *TOP1 TOP2 TopA+* cells. (**C–F**) DNA supercoiling and knotting of the 2-micron plasmid (C and D) and the YEp24 minichromosome (E and F) in *Δtop1 top2–4* and in *Δtop1 top2–4 TopA+* cells. Cells were sampled at 26°C (lane 1) and following 120 min at 37° (lane 2). (−)S and (+)S, negatively and positively supercoiled DNA; Lk, linking number topoisomers; *Kn*, knotted DNA forms; N, nicked DNA circles; L, linear DNA molecules. (**G** and **H**) *P^Kn^* of 2-micron and YEp24 in the conditions analyzed in panels (C) to (F). Data in panels (B), (G) and (H) are presented as mean ± SD of three experiments.

In previous studies, we showed that no significant changes of *P^Kn^* occur in *Δtop1* or *top2–4* cells, at either 26 or 37°C (27). For both these single mutants, since the action of topo II or topo I alone suffices to relax both (+)S and (−)S, the amount of transcriptional supercoiling is the same as *TOP1 TOP2* cells and so is the DNA knotting probability. Therefore, it is not likely that the boost of DNA knot formation observed in *Δtop1 top2–4 TopA+* cells is an artifact due to manipulation of cellular topoisomerases. To discard the possibility that the increase of DNA knotting could be a product of the exogenous *TopA* activity, we examined knot formation in *TOP1 TOP2 TopA+* cells sampled at 26°C and following 120 min at 37°C (Figure 3A). In either condition, (+)S did not occur and *P^Kn^* was about 0.02, similar to that observed in *TOP1 TOP2* cells (27) (Figure 3B). Likewise, to exclude the possibility that the increase of knot formation could be a singularity of YRp4, we examined the effect of (−)S and (+)S on DNA knot formation in other chromatin constructs, such as the 2-micron circle, an endogenous 6.3-kb plasmid of *S. cerevisiae* (Figure 3C and D), and YEp24, a 7.6-kb circular minichromosome (Figure 3E and F). In all cases, *P^Kn^* did not change significantly with (−)S, but increased about 10-fold with (+)S (Figure 3G and H).

**Figure 4.**
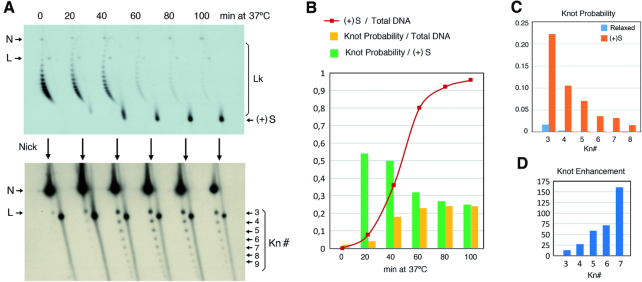
Correlation of (+)S with knot probability and complexity. (**A**) DNA supercoiling and knotting of YRp4 in *Δtop1 top2–4 TopA+* cells sampled at different time points (min) after shifting the cultures to 37°C. (**B**) Comparison of the accumulation rate of (+)S (red), *P^Kn^* values relative to total DNA (yellow) and *P^Kn^* values relative to the fraction of (+)S DNA (green). (**C**) Probability of individual knot populations (*Kn*# 3–8) in cells sampled at 0 min (relaxed chromatin, blue) and after shifting them to 37°C for 100 min ((+)S chromatin, orange). (**D**) Enhancement of individual knot populations shown in panel (C) (*Kn*# 3–7) upon accumulation of (+)S.

To substantiate further the correlation of (+)S and knot formation, we compared the accumulation rate of (+)S molecules with that of knotted molecules by sampling the cells at different time points after inducing topo II inactivation (Figure 4A). DNA knot formation increased rapidly as soon as (+)S molecules started to appear, not before. However, whereas the accumulation of (+)S molecules continued until it became nearly complete after 100 min, the accumulation of DNA knots reached a plateau (*P^Kn^* of ∼0.2) after 40–60 min of inducing topo II inactivation (Figure 4B, yellow bars). These distinct accumulation rates reflect the different mechanisms involved in DNA supercoiling and DNA knotting in the minichromosomes. Namely, upon inducing topo II inactivation, DNA transcription and relaxation of (−)S by *TopA* continue until virtually all minichromosomes are (+)S. Conversely, since *P^Kn^* values result from the DNA knotting/unknotting balance produced by topo II, they change only as long as there is residual topo II activity. Consequently, *P^Kn^* values stop increasing once topo II inactivation is complete. Since after 20–40 min of inducing topo II inactivation, the increased amount of knotted DNA molecules was nearly half the amount of (+)S molecules, the actual *P^Kn^* in (+)S chromatin was ∼0.5 (Figure 4B, green bars). Therefore, the residual activity of topo II, assuming it to be the same for the wild-type and the thermosensitive mutant, increased 25-fold the basal *P^Kn^* of intracellular DNA with accumulated (+)S.

We examined next whether the burst of DNA knot formation during (+)S of chromatin also changed the complexity of the knots. Both in relaxed and in (+)S chromatin, the knot 3_1_ was the most abundant form followed by knot populations that gradually diminished as their *Kn*# increased (Figure 4C). However, the enhancement of individual knot populations in (+)S markedly increased with knot complexity. The probability of knot 3_1_ increased ∼12 times, that of knot 4_1_ ∼25 times and that of knots 5_1_ + 5_2_ over 60 times (Figure 4D). Therefore, (+)S increased both knot formation and knot complexity.

### Transcriptional supercoiling of DNA in *TOP1 TOP2* cells also increases DNA knot formation

**Figure 5.**
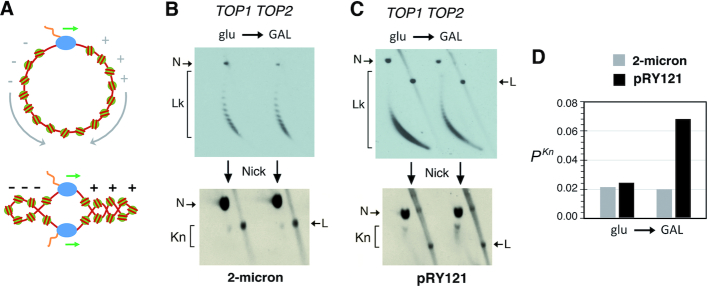
Transcriptional supercoiling increases knot formation in *TOP1 TOP2* cells. (**A**) Cancellation of the (+)S and (−)S waves generated by DNA transcription in circular minichromosomes (top) is precluded during bidirectional transcription, which produces separated (+)S and (−)S domains (bottom). (**B** and **C**) DNA supercoiling and knotting of the 2-micron plasmid (B) and the pRY121 minichromosome (C), which carries the *GAL1–GAL10* divergent promoter. *TOP1 TOP2* cells containing 2-micron and pRY121 were sampled during exponential growth at 26°C in glucose-containing media (glu) and after shifting them for 3 h in galactose-containing media (GAL); Lk, linking number topoisomers; *Kn*, knotted DNA forms; N, nicked DNA circles; L, linear DNA molecules. (**D**) *P^Kn^* of 2-micron and pRY121 in the conditions analyzed in panels (B) and (C).

Since (+)S is normally generated in front of the transcribing complexes, our results suggested that topo II-dependent DNA knotting should also increase during normal transcription in *TOP1 TOP2* cells. However, in our previous studies, we observed not only that transcription did not increase DNA knot formation, but also that it actually reduced the *P^Kn^* values of the minichromosomes (27). These observations can be reasoned by considering that the lifetime of the transcriptional supercoils produced in the circular minichromosomes is probably extremely short. Not only are the transcriptional supercoils rapidly relaxed by cellular topoisomerases in *TOP1 TOP2* cells, but also the (+)S and (−)S waves quickly cancel each other at the opposite side of the transcribing complex in these circular constructs (Figure 5A). The transient wave of (+)S may thereby be too brief to establish a significant boost of topo II-mediated DNA knotting and alter the *P^Kn^* values of the total population of minichromosomes. Basal *P^Kn^* values could instead diminish due to the unfolded state of transcriptionally active chromatin (27).

The above premises led us to hypothesize that the transient increase of DNA knotting during DNA transcription in *TOP1 TOP2* cells could be perhaps detected in circular minichromosomes by preventing the cancellation of transcriptional supercoiling waves. To this end, we analyzed DNA knotting in a minichromosome (pRY121), in which high rates of bidirectional transcription are induced from the galactose-inducible *GAL1–GAL10* divergent promoter (29). In this minichromosome, high transcription rates preclude quick relaxation of DNA by cellular topoisomerases, whereas bidirectional transcription prevents the cancellation of (+) and (−) supercoiled domains (Figure 5A). We found that shifting *TOP1 TOP2* cells containing pRY121 from glucose- to galactose-containing media did not alter the basal DNA knot probability of the endogenous 2-micron plasmid (Figure 5B). However, the DNA knot probability of pRY121 increased ∼3-fold following the galactose induction (Figure 5C and D). Remarkably, this increase of knot formation occurred with no net accumulation of (+)S or (−)S (Figure 5C), in agreement thus with the coexistence of twin supercoiled domains (Figure 5A). These results indicated that the boost of DNA knotting observed upon accumulation of (+)S in *Δtop1 top2–4 TopA+* can also occur during normal transcriptional supercoiling of DNA in *TOP1 TOP2* cells.

### DNA knots increase due to chromatin compaction driven by (+)S

*In vitro* studies have shown that topo II produces abundant and complex knots when DNA molecules are compacted (35–37). *In vitro* and *in vivo* studies have also indicated that (+)S rapidly compacts nucleosomal fibers (18,38,39). Therefore, we hypothesized that the burst of DNA knots observed *in vivo* was consequent to chromatin compaction driven by (+)S. In this respect, computer simulations of polymer chains have been useful to study the effect of compaction on knot probability and complexity (40–42). To test our notion, we then conducted numerical simulations of knot formation in nucleosomal fibers and examined the effect of compaction.

**Figure 6.**
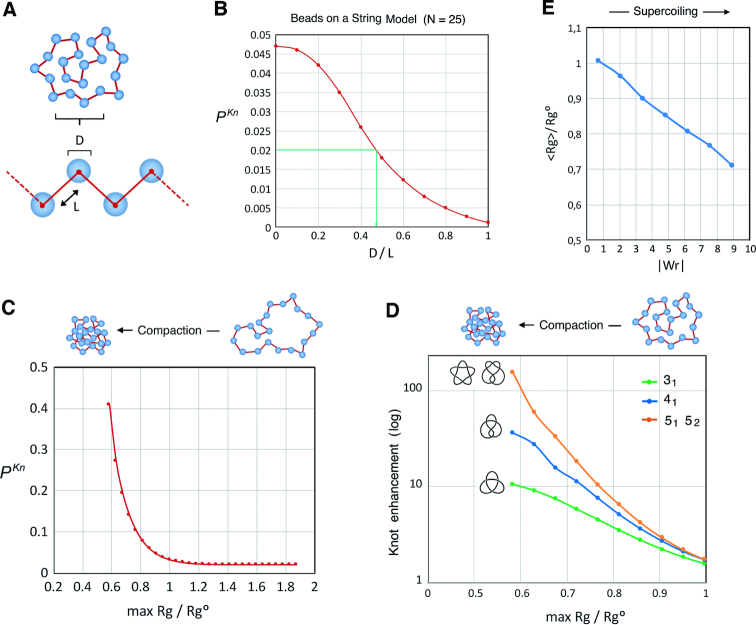
Computer simulations of the effect of chromatin compaction on DNA knot probability. (**A**) Beads-on-a-string model that simulates nucleosomal fibers. Beads of diameter *D* are connected by straight infinitely thin linkers of length *L*, such that the centers of two consecutive beads have a distance *D* + *L*. (**B**) Knot probability **(***P^Kn^*) of random configurations of rings of 25 beads as a function of *D/L*. The *P^Kn^* of 0.02 experimentally observed in unconstrained minichromosomes containing ∼25 nucleosomes is interpolated and matches *D/L* = 0.47. (**C**) Effect of compaction of knot probability. *P^Kn^* values of the reference model of the nucleosomal fiber (*N* = 25, *D/L* = 0.47) are plotted as a function of the gyration radius (*R_g_*) of the random configurations. Each point computes the *P^Kn^* of those configurations of *R*_g_ below a cutoff value (max *R*_g_) relative to the average gyration radius of the entire distribution of conformers (*R*_g_^0^). (**D**) Enhancement of individual knot populations by the effect of compaction. The enhancement of knots 3_1_, 4_1_ and 5_1_ + 5_2_ is computed for configurations of *R*_g_ below a cutoff value (max *R*_g_*/R*_g_^0^). (**E**) Reduction of *R*_g_*/R*_g_^0^ as a function of the absolute writhe (|*W*r|). For each sampled conformation, *R*_g_*/R*_g_^0^ was computed and averaged for different (binned) ranges of |*W*r|.

To obtain a representative beads-on-a-string model of the nucleosomal fiber, we generated random conformations of rings of *N* beads (nucleosomes) of diameter *D* connected by straight infinitely thin segments of length *L* (DNA linker) (Figure 6A). We then computed the knot probability of rings of 25 beads as a function of *D/L*. We found that the basal *P^Kn^* of ∼0.02 observed *in vivo* in unconstrained minichromosomes containing ∼25 nucleosomes (e.g. YRp4) was matched by a *D/L* ratio of 0.47 (Figure 6B). We next used this reference model of the nucleosomal fiber to generate millions of random conformers and profiled this unbiased sample in terms of the impact of compaction on knot probability. To this end, we set various cutoff values for the gyration radius (*R*_g_) of the configurations and computed knot probabilities considering only those with a lower *R*_g_ (max *R*_g_). We then plotted the knot probability obtained for max *R*_g_ values relative to *R*_g_^0^, the gyration radius of the unconstrained distribution of conformers (Figure 6C). As expected, *P^Kn^* values increased dramatically with increasing compaction. To reach the *P^Kn^* of ∼0.5 produced by (+)S *in vivo, R*_g_ had to be reduced more than 60% relative to *R*_g_^0^, corresponding thus to a 5-fold volume compaction. We calculated next the enhancement of individual knot populations (3_1_, 4_1_ and 5_1_ + 5_2_) produced by increasing compaction (Figure 6D). Akin to what was observed *in vivo*, the more complex a knot population the higher was its enhancement. Moreover, the enhancements of knots 3_1_, 4_1_ and 5_1_ + 5_2_ produced by (+)S *in vivo* (12, 25 and 60 times, respectively) all matched compression levels similar to those needed to increase *P^Kn^* to 0.5 (i.e. a max *R*_g_ of ∼60% of *R*_g_^0^). The simulation thus reproduced fairly well the spectrum of experimentally observed knots.

Finally, we asked how the compaction levels inferred from the simulation would correlate to DNA supercoiling. As an approximation to this problem, we computed the reduction of the radius of gyration of the conformers containing 25 beads (<*R*_g_>*/R*_g_^0^) as a function of their absolute writhe (|*W*r|) (Figure 6E). *R*_g_ reduction correlated to increasing values of |*W*r|, and the compaction levels needed to obtain the knot spectrum induced by (+)S *in vivo* involved |*W*r| values ≥9. These high Wr values are reachable *in vivo*, considering that the Lk of YRp4 increases more than 40 units when the minichromosome accumulates (+)S, and that this Lk difference translates mostly into changes of Wr (9). The overall simulation data thus supported that the basal DNA knotting probability of minichromosomes (*P^Kn^* 0.02) increases ∼25-fold (*P^Kn^* 0.5) as their nucleosomal fibers become compacted (a 5-fold volume reduction) during (+)S of DNA.

## DISCUSSION

The burst of DNA knots induced by (+)S in intracellular chromatin was not anticipated by previous theoretical models of the effect of supercoiling on knot formation and resolution. Computer simulations of polymer chains predicted that DNA supercoiling would inhibit DNA knotting (43). Supercoiling was also expected to facilitate DNA unknotting by topoisomerases by tightening the tangled regions (44–46) or confining them over biologically relevant timescales (44,47). Other mechanisms irrespective of DNA supercoiling were also expected to minimize knot formation in intracellular DNA. Topo II could use its *in vitro* capacity to reduce DNA knotting probability to levels below that of equilibrium conformations (48). DNA tracking motors, such as polymerases and condensins, could push and tighten DNA knots to facilitate their removal by topoisomerases (49). Clearly, none of these mechanisms appear to be effective to prevent the observed burst of DNA knots.

The alternative and simplest explanation for the increase of DNA knot formation is that (+)S compacts the nucleosomal fiber. In this respect, since interphase chromatin has a scaling behavior not dissimilar to that of a fractal globule (50,51), there is very little intermingling and so possible entanglements of DNA across topologically associating domains (TADs) and other high-order folds of chromatin (52,53). However, this is not the case within the length scales of nucleosomal fibers. Intramolecular DNA segments come in close proximity when nucleosome arrays are compacted by supercoiling or other mechanisms, thus increasing the incidence of DNA juxtapositions and thereby the chance that the DNA cross-inversion activity of topo II leads to DNA knots. Supporting this notion, topo II produces *in vitro* abundant and complex knots when DNA is compacted by supercoiling or other condensing agents (35–37). Likewise, numerical simulations demonstrated that the knotting probability and complexity of polymer chains largely increase by the effect of compaction (40–42). Our study extended these simulations of knot formation into a simplified model of the nucleosomal fiber. The results support that intracellular DNA knots are the statistically inevitable outcome of topo II activity, and show that the 25-fold increase of *P^Kn^* and the knot spectrum induced by (+)S can be reproduced by reducing to 60% the radius of gyration of the nucleosomal fibers, which corresponds to a 5-fold volume compaction.

The burst of DNA knot formation as a consequence of chromatin compaction also accounts for the differential effects of (+)S and (−)S of DNA. *In vitro* studies have shown that DNA over-twisting compacts nucleosomal fibers quicker and further than DNA untwisting (38,39). *In vivo* studies have also indicated that chromosomal domains under (+)S are more compacted than those under (−)S (18). This asymmetry in the conformational response of chromatin to helical tension of DNA has already explained why topo II is more proficient in relaxing (+)S than (−)S *in vivo* (9,19). The possibility that (−)S was inhibiting DNA knotting by means of other mechanisms then seems unlikely. For instance, (−)S could promote the formation of extended RNA/DNA hybrids (54), which could preclude the activity of topo II (55). In this respect, our previous studies indicated that there are no R-loops or other molecular interactions stabilizing the (−)S accumulated in the minichromosomes (19). The different effects of (+)S and (−)S on DNA knot formation thus corroborate that (+)S compacts intracellular chromatin to a much larger extent than (−)S. DNA knot analysis thereby proved to be very revealing about the elusive architecture of chromatin *in vivo*.

**Figure 7.**
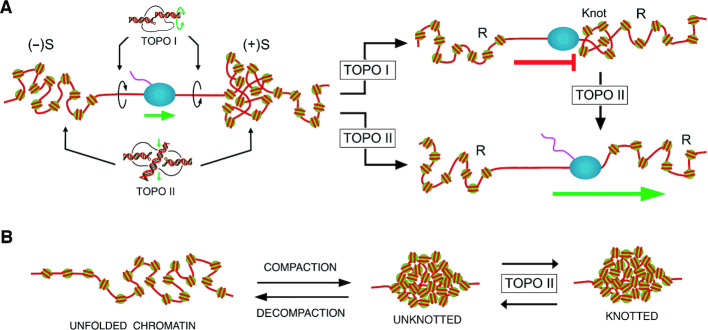
Implications of DNA knotting during gene transcription and chromatin compaction. (**A**) During normal DNA transcription, (+)S compacts chromatin and increases topo II-mediated knotting of DNA. Upon relaxation of (+)S, topo II dissolves DNA knots and transcription can continue. However, since topo I is not able to unknot DNA, the RNA polymerase is stalled by DNA knots when topo II fails to remove them. (**B**) Topo II-mediated knotting of DNA could be regulated to stabilize different conformational or compaction states of chromatin.

The (+)S in the circular minichromosomes examined in our study is driven by DNA transcription (1,2). Eukaryotic RNA polymerases transcribe DNA at rates of ∼100 bp/s, which means that DNA becomes over-twisted ∼10 turns/s (56). The degree of (+)S attained in circular minichromosomes (supercoiling density > +0.04) thus denotes the lower limit against which the transcription machinery is able to elongate *in vivo* (9). This capacity of RNA polymerases to confront (+)S may be crucial to transcribe DNA along native chromosomes, in which twin supercoiling domains cannot be cancelled as in the case of circular minichromosomes. Moreover, high levels of (+)S may occur when transcribing complexes encounter twist diffusion barriers or converge with other transcribing units or replication forks. Topo II-mediated knotting of DNA in native chromosomes might then reach levels comparable to those observed in the circular minichromosomes. In normal conditions, though, the occurrence of these knots is likely to be short-lived because, as soon as (+)S is relaxed or dissipated, topo II activity must restore the basal knotting probability of intracellular chromatin. However, if (+)S levels remain elevated or topoisomerase activity is altered, DNA knots could then persist and obstruct DNA transcription and chromatin assembly, as it has been demonstrated *in vitro* with knotted DNA templates (26,57). Remarkably, this scenario could explain why inactivation of topo II in *TOP1 top2-ts* yeast cells produces the stalling of RNA polymerases during the transcription of long genes (25), and why this stalling is rescued by exogenous expression of type-2, but not type-1 topoisomerases that relax supercoils but cannot remove DNA knots (Figure 7A). The concurrence of DNA supercoiling and knotting might also account for the effects of topoisomerase dysfunction during the transcription of long genes in mammal cells (20–24). This dark side of the topo II activity should therefore be taken into account when interpreting structural and functional alterations of intracellular chromatin.

As in the case of transcriptional supercoiling, our results highlight that other processes that compact chromatin might concur with topo II-mediated knotting of the embedded DNA. In this respect, an interesting possibility is that DNA knotting might be exploited to stabilize specific chromatin conformations. Previous studies indicate that mitotic chromosomes are shaped by topo II-sensitive DNA entanglements (58), and that topo II activity is required for both resolution and formation of facultative heterochromatin (59). DNA knot formation and removal could operate, for instance, to lock and unlock conformational states of chromatin (Figure 7B). Future research will uncover whether intracellular DNA knots are the only statistically inevitable outcome of topo II activity or whether DNA knot formation is also actively regulated. So far, we have uncovered that the DNA knotting probability changes dramatically with chromatin dynamics. Therefore, in addition to removing DNA supercoils and replication intertwines, topo II plays a crucial role in setting the DNA knotting–unknotting homeostasis of eukaryotic chromatin.
